# Serum concentration of zinc, copper, iron, and its associated factors among pregnant women of small-scale farming in western Ethiopia

**DOI:** 10.1038/s41598-023-30284-w

**Published:** 2023-03-14

**Authors:** Tariku Neme Afata, Seblework Mekonen, Gudina Terefe Tucho

**Affiliations:** 1 Department of Environmental Health Science and Technology, Jimma University, PO box 373, Jimma, Ethiopia; 2 Dambi Dollo Teachers College, Dambi Dollo, Oromia Region Ethiopia; 3 Department of Water and Public Health, Ethiopian Institute of Water Resources, Addis Ababa University, Addis Ababa, Ethiopia

**Keywords:** Biomarkers, Diseases, Health care, Risk factors

## Abstract

Micro-nutrients are required in small amounts to maintain growth and cell functioning to complete the life cycle through reproductions. However, pregnant women in developing countries like Ethiopia are vulnerable to multiple micro-nutrient deficiencies. Therefore, this study aimed at assessing the serum level of metals and associated factors like dietary diversity, and health-related problems in pregnant women among small-scale farming in Kellem Wellega, western Ethiopia. A cross-sectional laboratory-based study was conducted from June to August 2021 on 417 pregnant women attending antenatal care at rural healthcare facilities. Data was collected by using per-tested structured questionnaires via face-to-face interviews. The data analysis was conducted using SPSS version 24.0, and multivariate logistic regression analysis was performed to determine the association between predictor and outcome variables. A statistically significant was considered at *p* value < 0.05 for all the analyses. Our study findings showed that 62.1, 80.9, and 71.9% of the participants were deficient in iron, zinc, and copper micro-nutrient levels, respectively. Pregnant women who could not have formal education were 3.24 (AOR = 3.24, 95% CI 1.33–7.91) and 3.98 (AOR = 3.98, 95% CI 1.2–13.15) times more likely to show zinc and copper deficiency than those who attended secondary school and above, respectively. Furthermore, pregnant women involved only in farming activities were 0.57 (AOR = 0.57, 95% CI 0.36–0.91) and 4.33 (AOR = 5.72, 95% CI 2.34–13.97) times more likely to be exposed to iron and zinc deficiency than those who were engaged in other activities. This study revealed that pregnant women with low income were 6.36 times more likely to be exposed to zinc micro-nutrient deficiencies than those with high-income participants (AOR = 6.36, 95% CI 1.47–27.61). Additionally, those participants who ate a varied diet between 1 and 4 items per day were 2.26 (AOR = 2.26, 95% CI 1.43–3.59) and 2.77 (AOR = 2.77, 95% CI 1.6–4.61) times more likely to suffer zinc and copper micro-nutrient insufficiency than those who consumed 5–10 items per day. Finally, pregnant women who developed diarrhea in the past three months were 1.82 (AOR = 2.77, 95% CI 1.14–2.92) and 2.52 (AOR = 2.52, 95% CI 1.3–4.91) times more likely to be exposed to iron and copper deficiency than those who never show the symptoms, respectively. This study identified low concentrations of zinc, iron, and copper in the blood serum of pregnant women of small-scale farmers.

## Introduction

Micro-nutrients are vital for health and are required to maintain growth and cell functioning and complete the life cycle through reproduction^1^. Currently, the public health consequences of micro-nutrients are the most prominent. Inadequate dietary intake, lack of food availability, inequitable food distribution within the same household, lack of understanding of the importance of dietary diversity, and the frequent incidence of infectious diseases make pregnant women vulnerable to micro-nutrient deficiencies^2,3^. Micro-nutrient deficiencies are a major public health concern in many low and middle-income nations^4^.

Around 1.7% of the fatalities of pregnant women were caused by zinc deficiency^5^, and anaemia affects more than 30% of the global population, with iron deficiency being the most common cause of anaemia^6^. Zinc is an essential micro-nutrient that plays a vital role in developing, and reproducing, the immune system, gene expression, and maintaining cell organ integrity^7^. Furthermore, it is necessary for more than three hundred enzyme syntheses required to degrade carbohydrates, lipids, proteins, and nucleic acids^8^.

Zinc deficiencies can negatively affect the mother and foetus, causing complicated birth outcomes and increasing maternal morbidity, hypertension, fetal neural tube defects, low birth weight^9^, and poor neurobehavioral and cognitive development^10^. It also causes growth retardation, stillbirths, postpartum haemorrhage, insufficient uterine contraction, and abnormalities in gene replication^11^.

Iron is used to make extra blood (haemoglobin) for mothers and infants during pregnancy and helps oxygen move from the lungs to the rest of our body^12^. Getting not enough iron can prevent a condition of too few red blood cells that can make you feel tired and cause your baby to be born too small or too early^13^. Iron deficiency is associated with morbidity and mortality of the growing fetus, premature births, low birth weight, cognitive impairment, and death^14^.

Copper is an essential nutrient whose requirement is increased during pregnancy and lactation^15^ and it helps baby's heart formations, blood vessels and skeletal systems^16^. It is also an essential co-factor for various enzymes involved in biological processes^17^. Copper deficiencies impact physiologic systems such as bone marrow haematopoiesis, neurological system, and premature and low-birth-weight infants^18^.

Similarly, there was a significant public health issue regarding the micro-nutrient deficiencies of zinc, iron, and copper in pregnant women in Iran^19^, Vietnam^20^, and Nepal^21^. According to a systematic review study, pregnant women in Ethiopia, Kenya, and Nigeria had a zinc deficiency rate of 56, 70, and 46%, respectively. Anaemia was also common in those three countries, with rates ranging from 32 to 62%^22^. This is a moderate to a severe public health problem as per the WHO criteria^23,24^. Based on the EDHS 2017 report of Ethiopia, the maternal mortality rate has been estimated to be 401 per 100,000 live births due to a lack of micro-nutrients, and improper practice cause complications such as undernourishment and non-contagious diseases^25,26^.

Furthermore, the long rain season, high altitude, and agrochemical use for several years increase the loss of micro-nutrients from the soil and plants^27^. Additionally, dietary diversity, feeding practices, socioeconomic inequalities, and the prevalence of infectious diseases all have a significant role in the development of micro-nutrient deficiencies. As a result, micro-nutrient deficiencies are now recognized as serious public health issues, and there are related risk factors for low-level concentration. However, there is no regionally representative figure on the zinc, iron, and copper micro-nutrient concentration among pregnant women of small-scale farmers in Kellem Wellega zone of western Ethiopia. Therefore, the present study aimed to determine serum levels of zinc, iron, copper, and their associated factors among pregnant women of small-scale farming in western Ethiopia.

## Results

### Socio-demographic characteristics of respondents

From a total of 423 respondents, 417 of them were involved in this study giving a response rate of 98.6%. The mean age of the participants was 26.74 ± 5.6 years, with the minimum and maximum ages of 17 and 39 years. The average family size was 5.06 ± 2.72 with a minimum of 1 and a maximum of 14 persons. According to the findings, 60.4% of the respondents were farmers, while 39.6% were farming plus other activities. About 21.3% of the respondents could not have formal education, and nearly half (47.2%) of them attended primary school education. Most of the respondents were protestant (56.4%) religious followers, and 93.5% were earning a monthly income of less than 1000 Ethiopian birr. Furthermore, from the chi-square table age, ethnicity, religion, marital status, level of education and monthly income were significant associations with iron, zinc, and copper micro-nutrient deficiency (Table 1).

**Table 1:** Socio-demographic factors of pregnant women in Kellem Wellega zone, Western Ethiopia in 2021

Explanatory variables (%)	F (%)	Iron in µgL^−1^			Zinc in µgdL^−1^			Copper in µgdL^−1^		
		ND ≥ 10	Deficient < 10	χ^2^	ND ≥ 51	Deficient < 51	χ^2^	ND ≥ 70	Deficient < 70	χ^2^
*Age in years*										
< 25	35.5	51	98		27	122		51	98	
25–35	56.6	93	143		56	180		93	143	
> 35	7.7	14	18	0.46	0	32	0.05*	14	18	0.01*
*Ethnicity*										
Oromo	87.1	130	233	0.52	83	270	0.001*	130	223	0.001*
Amara	12.9	23	31		0	54		23	31	
Others	2.4	5	5		0	10		5	5	
*Religion*										
Orthodox	21.8	23	68		25	66		23	68	
Protestant	56.4	93	142	0.01*	58	177	0.01*	93	142	0.3
Catholic	1.92	2	9		0	8		0	8	
Muslim	19.9	40	40		0	83		42	41	
*Marital status*										
Married	93.5	148	242	0.82	75	315	0.01*	105	285	0.04*
Single	2.61	4	5		0	9		4	5	
Divorced	3.12	5	8		8	5		4	9	
Windowed	1.2	1	4		0	5		4	1	
*Family size*										
< 5	67.1	104	176		59	221		104	176	
≥ 5	32.9	54	83	0.65	24	113	0.4	54	83	0.13
*Education*										
No formal educ	21.3	24	65	0.01*	12	77	0.001*	41	48	0.02*
Primary school	47.2	63	134		58	139		46	151	
Secondary school and above	31.4	71	60		13	118		30	101	
*Occupation*										
Farming	60.4	82	170	0.001*	191	61	0.01*	70	182	0.88
Farming and other activities	39.6	76	89		143	22		47	118	
*Income in Birr*										
< 1000_(19USD)_	52.5	90	129	0.72	38	181	0.01*	61	158	0.08
1000–2000_(19–38USD)_	33.8	54	87		101	40		103	38	
> 2000 _(38USD)_	13.7	14	43		52	5		39	18	

*χ*^*2*^ Chi square test, *ND* non-deficient, *Educ.* education, *F* frequency.

### Access to a diversified diet

**Figure 1:**
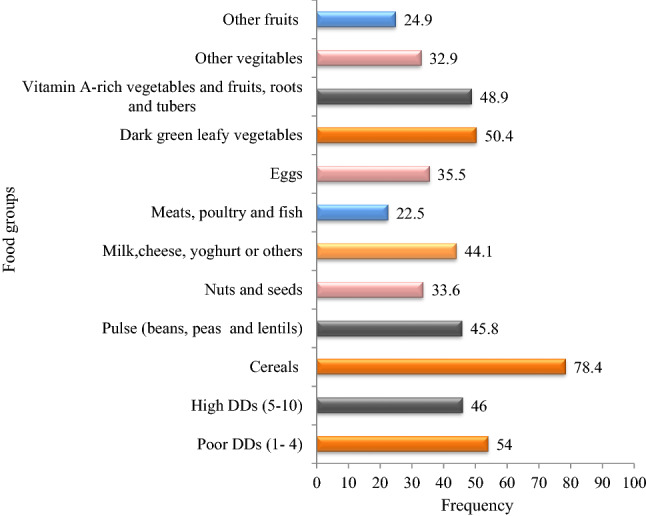
Dietary diversity score of pregnant women who ate each food type at least once a day in Kellem Wellega zone, western Ethiopia.

Most pregnant women consumed meals at any time that was convenient for the study participants, particularly after they returned home. Typically, they consume cereals or starch staple food (78.4%), dark green vegetables (50.4%), and Vitamin A-rich vegetables and fruits, roots, and tubers (48.9%) were the most commonly consumed food types among the study participants. Meats, poultry, and fish foods were the least food group accounting for a total consumption of 22.5%. The one-day dietary diversity survey shows that 225 (54%) of the pregnant women were categorized as having a low dietary diversity score (DDS, 1–4), and 192 (46%) as having a high dietary diversity score (DDS, 5–10) (Fig. 1).

### Health status of pregnant women

About 208 (49.9%), 335 (80.3%), and 208 (49.9%) pregnant women did not take any iron, iron-folic acid, or multivitamin tablets in the previous three months, respectively. The respondents have reported that 53 (36.7%) had diarrhea, 102 (24.5%) coughing, 65(15.6%) malaria, and 218 (52.3%) intestinal worms in the previous three months (Table 2).

**Table 2:** Clinical results among pregnant women attending antenatal care in Kellem Wellega zone, Western Ethiopia.

Characteristics of the respondents	Yes (%)	No (%)
During the last three months, did you take any iron tablets?	209(50.1)	208(49.9)
Did you have diarrhea or loss of appetite in the past three months?	153(36.7)	264(63.3)
During the last three months, did you take any multivitamin tablets?	82(19.7)	335(80.3)
Have you been ill with a cough or breathing problems for three months?	102(24.5)	315(75.5)
Have you been ill with malaria in the past three months?	65(15.6)	352 (84.4)
Did you take any drugs for intestinal worms in the past three months?	218(52.3)	199(47.7)

### Prevalence and actors associated with iron deficiency

From the serum analysis, 138 (37.9%) pregnant women have sufficient levels of iron, and 259 (62.1%).of the participants were deficient in these micro-nutrients. The mean serum iron concentration was (34.78 ± 41.09) μgdL^−1^. Moreover, variables like level of education, monthly income, pulse (nuts, lentils and legumes), vegetables, iron supplements, problems of diarrhea, and malaria have a *p* value less than 0.25 in bi variate analysis and were a candidate for multivariate logistic regression. However, due to potential con-founders, some variables were significant associations in the bi-variate analysis but their significance disappeared in the multivariate analysis. Thus, education, occupation, vegetables, iron supplements, and diarrhea were significant associations with iron micro-nutrient deficiency in multivariate logistic regression analysis.

Accordingly, pregnant women who could complete secondary school and above were 87% less likely to be exposed to iron deficiency than those who attended non formal education (AOR = 0.13, 95% CI 0.06–0.27) and participants who were only involved in other activities were 43% less likely to be exposed to iron deficiencies than those who were involved in only farming activities (AOR = 0.57, 95% CI 0.36–0.91).

On the other hand, pregnant women who ate vegetable items were 48% less likely exposed to the iron deficiency than those who didn’t consume vegetables (AOR = 0.52, 95% CI 0.31–0.85) and pregnant women who regularly took iron supplements had a significantly lower risk of iron deficiency than those who did not (AOR = 0.43, 95% CI 0.24–0.77). Furthermore, pregnant women who developed diarrhea or loss of appetite in the past three months were 1.82 times more likely to be exposed to the iron deficiency than those who never show the symptoms (AOR = 1.82, 95% CI 1.14–2.92) (Table 3).

**Table 3:** Results of logistic regression model for risk factors associated with iron deficiency of pregnant women small-scale farmers in Kellem Wellega zone, western Ethiopia, 2021.

Explanatory variables	Iron serum levels (μgL^−1^)		COR (95% CI)	AOR (95% CI)
	ND ≥ 10	Deficient < 10		
*Education*				
Non formal	24	65	0.31(0.18–0.56)	0.13(0.06–0.27)*
Primary school	63	134	0.40(0.25–0.63)	0.3(0.17–0.51)*
Secondary school and above	71	60	1	1
*Occupation*				
Farming	82	170	1.77(1.18–2.65)	0.57(0.36–0.91)*
Farming and other activities	76	89	1	1
*Income in birr*				
< 1000 _(19USD)_	90	129	2.14(1.11–4.15)	0.95(0.32–2.78)
1000**–**2000 _(19–38 USD)_	54	87	1.91(0.95–3.81)	2.34(0.80–6.83)
> 2000 _(38USD)_	14	43	1	1
*Pulse (Nuts, lentils and legumes)*				
No	104	122	0.46 (0.31–0.7)*	1.51(0.93–2.47)
Yes	54	137	1	1
*Vegetables*				
No	71	139	1.42 (0.95–2.11)	0.52(0.31–0.85)*
Yes	87	120	1	1
*Iron supplements*				
Yes	94	115	1	1
No	64	144	1.84(1.23–2.75)	0.43(0.24–0.77)*
*Did you have diarrhea in the past three months?*				
Yes	74	79	0.5(0.33–0.75)*	1.82(1.14–2.92)*
No	84	180	1	1
*Have you been ill with malaria in the past three months?*				
Yes	29	36	0.72(0.42–1.23)	0.47 (0.21–1.07)
No	129	223	1	1

*AOR* adjusted odds ratio, *COR* crude odds ratio, *CI* confidence interval, Other activities: (daily labourer, private business, non-Government and civil servants), *ND* non deficient; and *represent the level of significance at *p* values less than 0.05.

### Prevalence and factors associated with zinc deficiency

From the serum analysis, 83(19.1%) of pregnant women have sufficient levels of zinc 334(80.9%).of the participants were deficient in these micro-nutrients. The mean serum zinc concentration was (30.32 ± 60.77) μgdL^−1^. Factors like level of education, occupation, monthly income, dark green leafy vegetables, meats, roots and tubers, cereals, milk, fruits, diversity of food consumed, problems of malaria, worms, and multivitamins have a *p* value less than 0.25 in bi variate analysis were a candidate for multivariate logistic regression. However, due to potential con-founders, some variables were significant associations in the bi-variate analysis but their significance disappeared in the multivariate analysis. Thus, the level of education, occupation, monthly income, vegetables, meats, roots and tubers, cereals, and dietary diversity (DD) of food were significant associations with zinc micro-nutrient deficiency in multivariate logistic regression analysis.

Accordingly, the pregnant women who have non formal education were 3.24 times more likely to be exposed to zinc deficiency than those who have a secondary school and above (AOR = 3.24, 95% CI 1.33–7.91). Pregnant women involved only in farming activities were 5.72 times more likely to be exposed to zinc deficiency than those who were only engaged in farming and other activities (AOR = 5.72, 95% CI 2.34–13.97).

This study revealed that pregnant women with low income were 6.36 times more likely to be exposed to zinc micro-nutrient deficiencies than those with high-income participants (AOR = 6.36, 95% CI 1.47–27.61). On the other hand, pregnant women who ate vegetable items (AOR = 4.22, 95% CI 1.87–9.50), meats (AOR = 0.02, 95% CI 0.01–0.05), roots and tubers (AOR = 8.34, 95% CI 3.14–22.13), and cereals (AOR = 8.4, 95% CI 3.52–20.05) were less likely exposed to zinc deficiency than those who did not consume. Furthermore, those participants who ate a varied diet between 1 and 4 items per day were 2.26 more likely exposed to zinc micro-nutrient insufficiency than those who consumed 5–10 items per day (AOR = 2.26, 95% CI 1.43–3.59) (Table 4).

**Table 4:** Results of logistic regression model for risk factors associated with zinc deficiency of pregnant women small-scale farmers in Kellem Wellega zone, western Ethiopia, 2021.

Explanatory variables	Zinc serum levels (μgdL^−1^)		COR (95% CI)	AOR (95% CI)
	ND ≥ 51	Deficient < 51		
*Education*				
Non formal	12	77	3.79(1.98–7.25)*	3.24(1.33–7.91)*
Primary school	58	139	1.42(0.61–3.26)	1.09(0.34–3.48)
Secondary school and above	13	118	1	1
*Occupation*				
Farming	61	191	2.08(1.22–3.54)	5.72(2.34–13.97)*
Farming and other activities	22	143	1	1
*Income in Birr*				
< 1000_(19 USD)_	38	181	4.12(1.53–11.06)*	6.36(1.47–27.61)*
1000–2000_(19_–_38 USD)_	40	101	2.18(0.82–5.83)*	4.49(1.07–18.79)*
> 2000 _(38 USD)_	5	52	1	1
*Dark green leafy vegetables*				
No	29	178	1	1
Yes	54	156	2.13(1.29–3.5)	4.22(1.87–9.50)*
*Meats*				
No	43	280	1	1
Yes	40	54	0.21(0.12–0.35)	0.02(0.01–0.05)*
*Roots and tubers*				
No	56	146	1	1
Yes	27	186	2.61(1.57–4.33)	8.34(3.14–22.13)*
*Cereals*				
No	34	66	1	1
Yes	49	268	2.82(1.67–4.71)	8.40(3.52–20.05)*
*DDS*				
Poor (1–4)	30	195	2.09(1.53–2.86)*	2.26(1.43–3.59)*
High (5–10)	49	143	1	1

*AOR* adjusted odds ratio, *COR* crude odds ratio, *CI* confidence interval; other activities: (daily labourer, private business, non-Government and civil servants), *ND* non deficient; and *represent the level of significance at *p* values less than 0.05.

### Prevalence and factors associated with copper deficiency

From the serum analysis, 117(28.1%) pregnant women have sufficient levels of copper 300(71.9%) of the participants were deficient in these micro-nutrients. The mean serum zinc concentration was (55.03 ± 71.22) μgdL^−1^. Factors like age, level of education, milk, meats, roots and tubers, fruits, dietary diversity of food consumed, problems of diarrhea, breathing problems, and intestinal worms have a *p* value less than 0.25 in bi-variate analysis and were candidates for multivariate logistic regression. However, due to potential con-founders, some variables were significant associations in the bi-variate analysis but their significance disappeared in the multivariate analysis. Thus, age, level of education, milk, meats, fruits, diversity of food consumed, problems of breathing problems, intestinal worms, and diarrhea were significant associations with copper micro-nutrient deficiency in multivariate logistic regression analysis.

Thus, the pregnant women whose age was greater than thirty-five years were 3.98 times more likely exposed to copper deficiency than those whose age was less than 25 years (AOR = 3.98, 95% CI 1.2–13.15). On the other hand, pregnant women who have no formal education were 2.64 times more likely to be exposed to copper deficiency than those who have a secondary school and above educational level (AOR = 2.64, 95% CI 1.22–5.68). Pregnant women who ate fruit items were less likely to be exposed to copper deficiency than those who did not consume fruits (AOR = 0.39, 95% CI 0.23–0.65), meats (AOR = 0.47, 95% CI 0.24–0.95), milk (AOR = 0.28, 95% CI 0.13–0.58). Furthermore, those participants who ate a varied diet between 1 and 4 items per day were 2.77 times more likely exposed to copper micro-nutrient insufficiency than those who consumed 5–10 items per day (AOR = 2.77, 95% CI 1.66–4.61). pregnant women who developed diarrhea or loss of appetite in the past three months were 2.52 times more likely to be exposed to copper deficiency than those who never show the symptoms (AOR = 2.52, 95% CI 1.3–4.91) (Table 5).

**Table 5:** Results of logistic regression model for risk factors associated with copper deficiency of pregnant women small-scale farmers in Kellem Wellega zone, western Ethiopia, 2021.

Explanatory variables	Copper serum levels (μgdL^−1^)		COR (95% CI)	AOR (95% CI)
	ND ≥ 70	Deficient < 70		
*Age in years*				
< 25	68	81	1	1
25–35	45	191	5.88(1.96–17.59)	16.35(4.76–56.1)*
> 35	4	28	1.65(0.55–4.94)	3.98(1.20–13.15)*
*Education*				
Non formal	41	101	2.88(1.61–5.15)*	2.64(1.22–5.68)*
Primary school	46	151	1.03(0.61–1.0.73)	0.72(0.38–1.35)
Secondary school and above	30	101	1	1
*Fruits*				
No	68	245	3.21(2.01–5.13)	0.39(0.23–0.65)*
Yes	49	55	1	1
*Meats*				
No	122	201	1.84(1.14–3.02)*	0.47 (0.24–0.95)*
Yes	36	58	1	1
*Milk, cheese and yogurt*				
No	92	141	0.72(0.47–1.12)	0.28(0.13–0.58)*
Yes	66	118	1	1
*Roots and tubers*				
No	56	146	1.56(1.01–2.40)*	0.61(0.33–1.13)
Yes	27	186	1	1
*Dietary diversity score (DDS)*				
Poor (1–4)	30	195	1.57(1.19–2.08)	2.77(1.66–4.61)*
High (5–10)	49	143	1	1
*Did you have diarrhea in the past three months?*				
Yes	74	79	0.50(0.5–1.2)	2.52(1.3–4.91)*
No	84	180	1	1
*Have you been ill with breathing problems for three months?*				
Yes	43	59	0.59(0.35–1.01)	0.29(0.11–0.77)*
No	115	200	1	1
*Did you take any drugs for intestinal worms in the past three months?*				
Yes	77	141	1.26(0.92–2.16)	0.44(0.24–0.8)*
No	81	118	1	1

*AOR* adjusted odds ratio, *COR* crude odds ratio, *CI* confidence interval, *ND* non deficient; and *represent the level of significance at *p* values less than 0.05.

## Discussion

**Figure 2:**
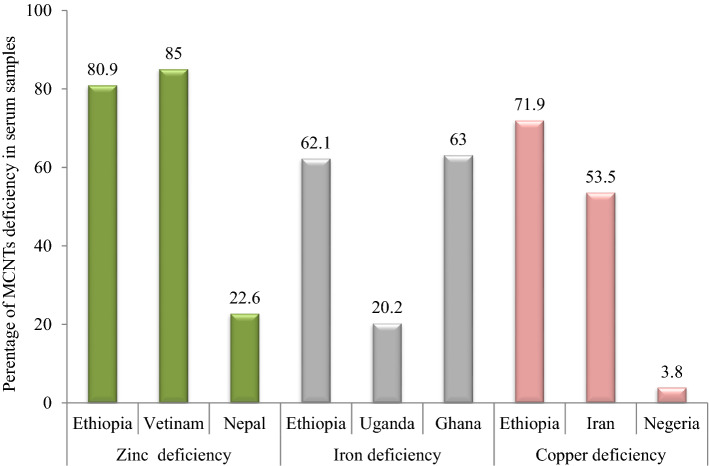
Prevalence of zinc, iron, and copper deficiency level (μgdL^−1^) of pregnant women in different countries.

The prevalence of zinc, iron, and copper deficiency for pregnant women in small-scale farmers of the study area was 80.9, 62.1, and 71.9%, respectively. According to the IZiNCG, micro-nutrient deficiencies are a public health concern if the prevalence is greater than 20%^28^. Thus, the study provides evidence for the public health concern of zinc, iron, and copper deficiencies and is in line with the study conducted in Iran^19^, Vietnam^20^, Nepal^21^, Uganda^29^, Ghana^30^, and Nigeria^31^ (Fig. 2).

The prevalence of zinc deficiency in the study area was higher than the studies conducted in southwest Ethiopia (55.3%)^32^, Nepal l (22.6%)^21^, and lower than that reported in Vietnam (85%)^20^. This discrepancy may be due to socioeconomic, geographical area, and consumption of cereal foods among pregnant women^33^. Moreover, pregnant women living in rural areas have high food insecurity, low intakes of foods of animal origin, lack of nutritional education, and instability in the study area^34^. Further, pregnant women from rural areas were more likely to be involved in a strenuous activity that makes high excretion of micro-nutrients. Furthermore, the study participants reported their infection with intestinal parasites, which might increase the loss of zinc through diarrhea and intestinal bleeding^35^. The present study revealed that 76% of pregnant women were highly dependent on the consumption of cereals diets that contain a high level of phytates, the most potent inhibitors of zinc absorption and bio-availability^5^. Furthermore, crops grown locally have zinc deficiency due to Ethiopian soil having a high pH value and poor organic matter that puts the households at risk of inadequate zinc intake^36,37^.

This study revealed that pregnant women with low income were 5 times more likely to be exposed to micro-nutrient deficiencies of iron and zinc serum than those with a high income. This means that increasing women's income is an essential strategy for reducing micro-nutrient deficiency and improving the health of pregnant women. Previous research found that pregnant women in Ethiopia, Kenya, and India who reported a lack of dietary diversity were considerably more likely to be from low-income families^38,39^. Moreover, in Ghana, those with a higher monthly income had significantly greater dietary diversity scores than those with a lower income^40^. At the same time, a study in China found that low income was connected to developing a micro-nutrient deficit^41^.

On the other hand, pregnant women consuming diverse diets between 5–10 were 63% less likely to be exposed to zinc micro-nutrient deficiency than those who consumed 1–4 various food per day. This finding aligns with the study conducted in northwest Ethiopia^34^. The possible justification could be related to the number of consumed foods with increased foods of animal sources, which are good sources of zinc. Hence consumption of diversified foods helps women get better-quality foods that enhance their immunity and improve their nutritional status^34^.

In this study, the overall prevalence of iron deficiency among pregnant women was 62.1%. The iron deficiency in the study area is higher than in a study conducted in Uganda (17.7%)^42^, Rwanda (29.4%)^43^, and lower than in Ghana(63%)^30^. This might be due to the difference in the study period, socioeconomic disparity, and geographical location of the study area. Educational status was significantly associated with the prevalence of anaemia among study participants. On the other hand, pregnant women who regularly took iron supplements had a significantly lower risk of iron deficiency than those who did not. Pregnant women who visit health centers during ANC and have not received iron supplements due to forgetfulness, lack of comprehensive knowledge on anaemia, and any information about the importance of iron supplementation during pregnancy are at a high risk of iron deficiency^44^. A study in Ethiopia revealed that 87.6% of pregnant women took iron-folic acid (IFA) below the recommended dose during the index pregnancy^45^.

The prevalence of copper deficiency in this population is 71.9%. This is relatively higher than that reported in Sudan (3.7%)^46^ and lower than in Iranian (86%)^47^. This is due to inadequate dietary intake, malabsorption, micro-nutrient interactions, infections, household food insecurity, nutrition knowledge, and consumption of diets high in phytates^31,35^. In the present study, a higher prevalence of copper deficiencies was found among women who often consumed carbohydrates and cereals. Less consumption of meat, fish, and vegetables may partly have contributed to the high Prevalence of copper deficiencies. Since copper is abundantly found in meat and vegetables^33^. The high prevalence rates of copper and zinc deficiency may also be partly attributed to its interactions with other divalent metal ions, such as iron, calcium, and magnesium^31^.

Finally, this study revealed that pregnant women who have not formal education were more likely to be exposed to zinc, iron, and copper micro-nutrient deficiencies than that in secondary school and above. This is supported by studies conducted in the southwest of Ethiopia^34^. This trend may reflect that women without formal education may be unaware of health and nutrition-related issues that jeopardize their health and nutritional conditions^48^. Moreover, women with a higher educational level are expected to have greater employment options and be wealthier, making them less vulnerable to food insecurity, creating more independent decisions, and accessing household resources that are crucial to their nutritional well-being^39^.

Moreover, pregnant women involved in farming and other activities like government or non-government work were less likely to be exposed to iron and zinc micro-nutrient deficiencies than those engaged in farming activities. This shows maternal occupations were directly related to the risk of micro-nutrient deficiency in pregnant women. The majority of pregnant women involved in farming operations are uneducated and belong to the poorest socioeconomic groups, which impacts their practice. It is expensive to purchase nutritionally adequate foods like mineral-rich foods like meat, eggs, fruits, and vegetables. Furthermore, many are so busy throughout the day that they miss meals, resulting in nutritional deficiency^39^.

### Strengths and limitations of the study

The strength of our study includes following strict aseptic techniques during blood drawing, transportation, and processing of blood samples. In addition, we used graphite furnace atomic absorption spectroscopy, which has a high sensitivity for measuring iron, zinc, and copper serum levels, to avoid underestimation. However, the study had certain limitations. Lucks representativeness of all districts and dietary diversity was measured using a 24-h dietary recall during pregnancy, which was dependent on memory and recall accuracy, such data tend to under or overestimate actual food consumption.

## Conclusions

This study concludes that the micro-nutrient deficiency of pregnant women small-scale farmers may be related to occupation, low level of education, low monthly income as well as not consuming cereals, root and tubers, vegetables, fruits, milk products, meats, eggs, and low diversity of food consumed by pregnant women small-scale farmers have a significant contribution to micro-nutrient deficiency of iron, zinc, and copper. Moreover, problems of diarrhea, coughing (breathing), malaria, and intestinal worms cause micro-nutrient deficiency in pregnant women small-scale farmers. It is recommended to improve the dietary diversity and consumption of animal food sources to minimize problems among the target groups. In light of our study, we recommend a further interventional study to confirm the cause-effect relationship between iron, zinc, and copper deficiency for pregnant women in the study area.

## Materials and methods

### Study setting

**Figure 3:**
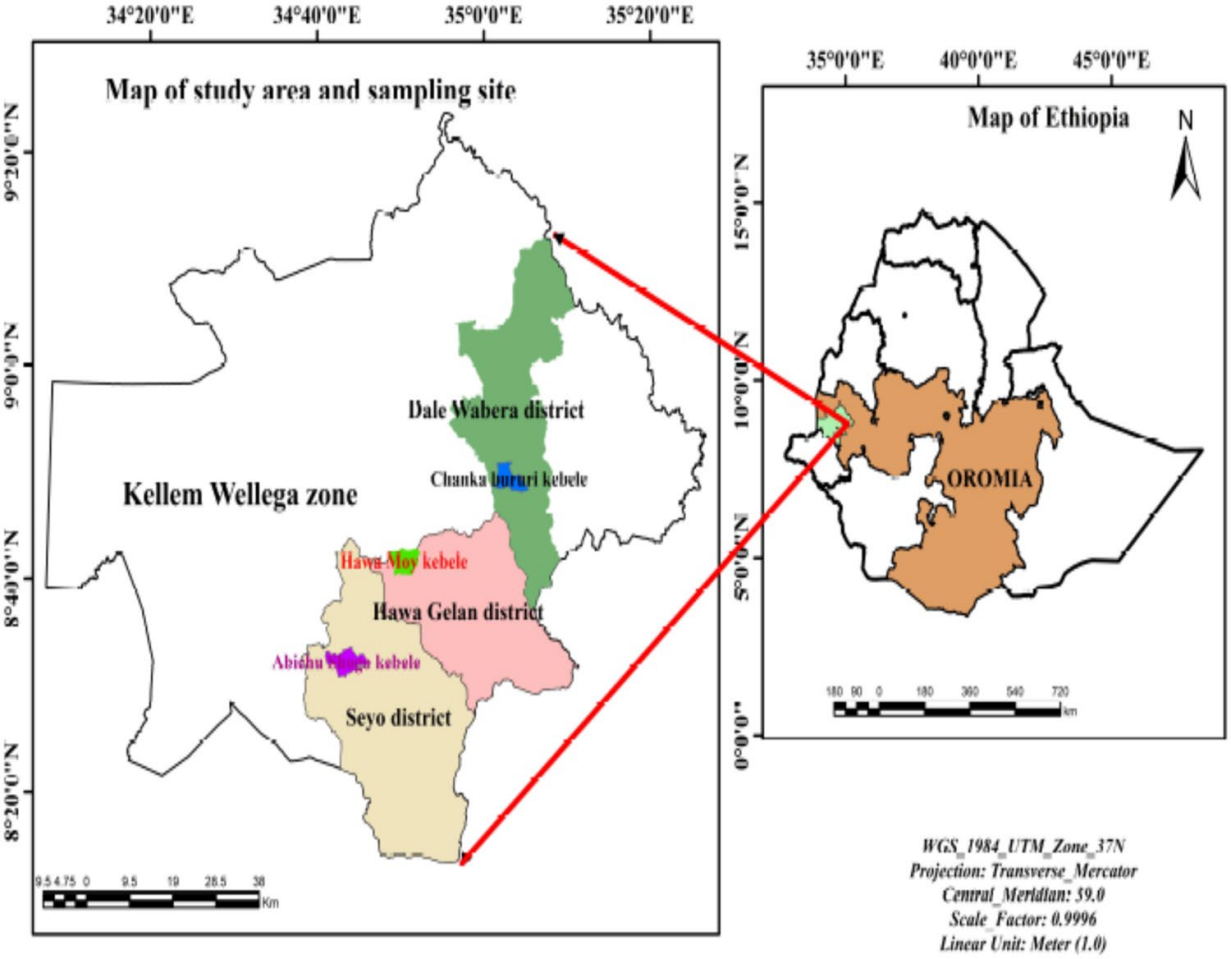
Map of the study area.

The study was conducted in Kellem Wellega zone of the Oromia region, western Ethiopia. The study area is 672 km away from Addis Ababa which is the capital city of Ethiopia. The zone is divided into ten districts, with a total population of 965,000, of which 49.75% are females^49^. Our study purposely selected three districts, such as Sayyo, Hawa Gelan, and Dale Wabara, due to the addition of high-yielding varieties, intensive cultivation systems, application of fertilizers for several years, and increased acidity of the soil in these areas^50^. The selected districts do not use much organic manure that can balance plant nutrients and increase their uptake by plants. The site is situated between 1701 and 1830 m above sea level. The weather pattern fluctuates from heavy summer rains to a brief rainy season to a dry winter. The yearly precipitation ranges from 800 to 1200 mm, with daily temperatures ranging from 15 to 25 °C^51^. The region is well-known for its enormous agricultural output, including coffee, maize, teff, wheat, barley, bean seed, and sorghum. The map of the study area is indicated in Fig. 3.

### Design of the study, population, and sampling techniques

A cross-sectional laboratory based study was conducted on pregnant women who attended antenatal care in selected health facilities independent of their stage of pregnancy and were asked to participate. After allocating participants to each of the three districts, the study participants were chosen using systematic random sampling techniques. The first study subject for each health centre was randomly selected by a lottery method followed by enrolling every second pregnant woman.

### Determination of sample size and its procedure

The sample size was obtained using a single population proportion formula considering a level of statistical significance at 95%, a 5% margin of error, a 55% proportion of micronutrient adequacy during pregnancy in Northwest Ethiopia^52^, and a 10% non-response rate. As a result, the sample size is calculated using the one-point sample estimate formula.

**Figure 4:**
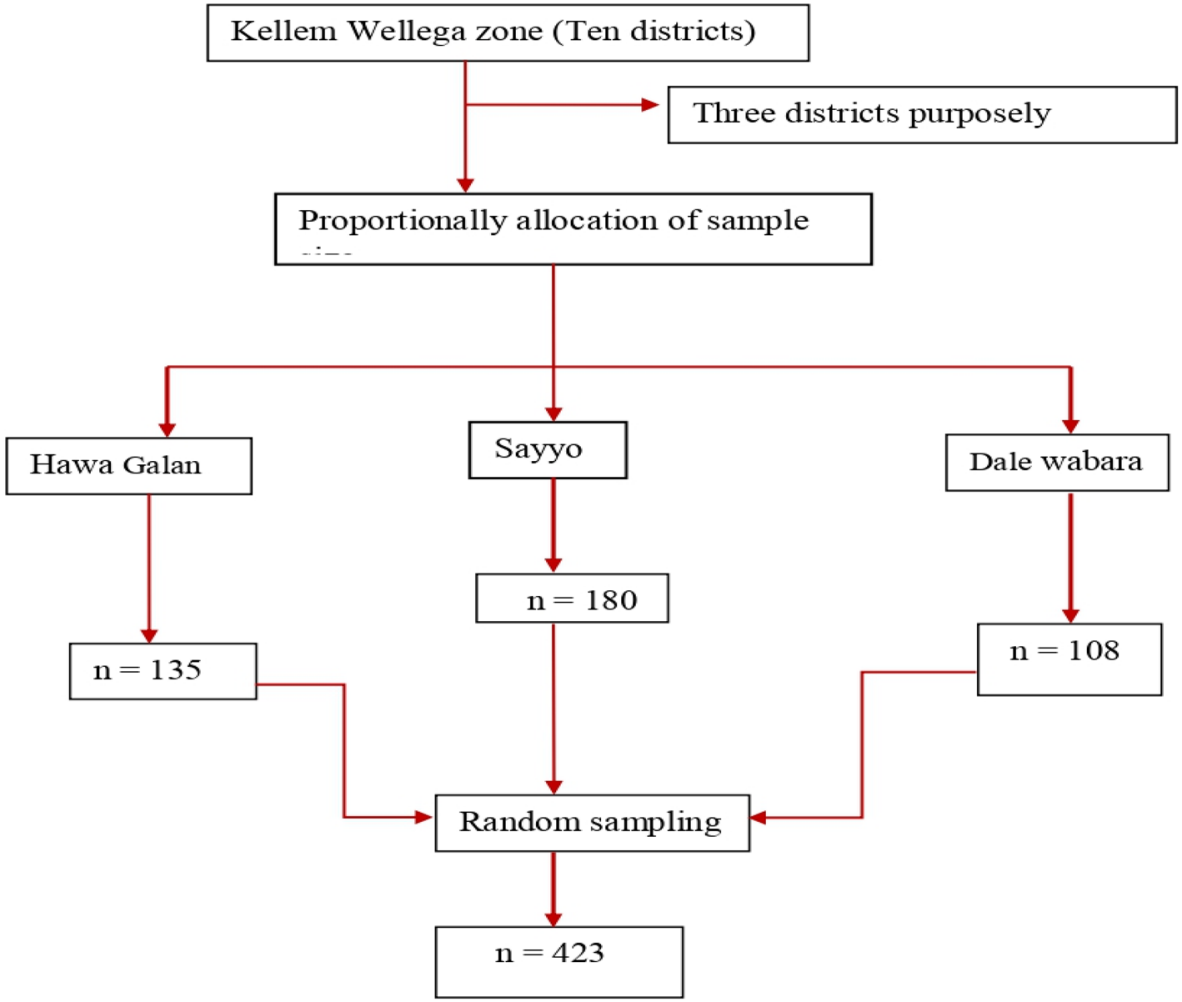
Schematic presentation of sampling techniques for pregnant women of Kellem Wellega zone in 2021.

${\text{n}} = \frac{{({\text{Z}}\alpha /2)^{2} \left( {\text{p}} \right)\left( {\text{q}} \right)}}{{({\text{SE}})^{2} }} = \frac{{(1.96)^{2} \left( {0.55} \right)\left( {1 - 0.55} \right)}}{{(0.05)^{2} }} = 384$, Four hundred twenty-two pregnant women in small-scale farmers were considered for the study after adding 10% (38.4 ≈ 39) for the non-response rate. The total sample size was four hundred twenty-three (423) were designed to participate in the study. Finally, proportionally allocating the sample size to the selected three districts was based on the preceding three months' daily client flow of the clinic, which was collected by reference client registration record before data collection, the average number of clients that attended the ANC clinic daily throughout the data collection period was approximated in Fig. 4.

### Inclusion and exclusion criteria

The study included pregnant women who had lived for at least six months in the Sayyo, Hawa Gelan, and Dale Wabara districts of the Kellem Wellega zone who had attended an ANC visit and were willing to participate. Those who were gravely ill and unable to answer throughout the data-collecting period were not included.

### Serum Sample and survey data collection

A standardized self-administered questionnaire was used to collect data at health centers. A standard questionnaire was prepared in English with minor changes, then translated into Afan Oromo for clarity, and then back to English to validate the accuracy of the translation. Three laboratory workers and three supervisors were involved in the data-gathering process. Data collectors and supervisors received one day of training. The supervisors assessed and checked the completed questionnaires for accuracy every day, and the data collectors received timely feedback the day before the procedure.

The dietary diversity score (ten questions) for pregnant women is appropriate nutrient intake^53^ and is one of the indicators for determining nutrient adequacy among pregnant women^54^. The dietary diversity score (DDS) was calculated using a 24-h recall method. According to the Food and Agriculture Organization (FAO) of the United Nations^55^, the degree of dietary diversity score was computed out of ten and classified as high (DDS, 5–10), and low (DDS, 1–4). Cereals, pulse (beans, peas, and lentils), (Nuts and seeds), (milk, cheese, yogurt, or others), (meats, poultry, and fish), eggs, dark green leafy vegetables, vitamin A-rich vegetables and fruits, roots and tubers, other vegetables and other fruits are among these food groups. The pregnant women were asked to recall all foods ingested the day before from the aforementioned food groups. Since the DDS appears to be a good measure of household micronutrient sufficiency.

Finally, about 5 mL of blood samples were collected by vein puncture from each participant in the morning time without eating their breakfast in a sterile vacationer tube. Then the blood samples were allowed to stand for 30 min at room temperature, and the coagulate was centrifuged in disposable sterile vacuum plastic tubes at 3000 rpm for 10 min, and the serum specimen was then separated, and repackaged into clean, metal-free, polypropylene tubes at − 20 °C for a short term. Subsequently, serum samples were shipped icebox to the Laboratory of Jimma University Environmental Health Science and Technology and stored in a deep freeze at − 20 °C until analysis.

### Assessing the level of iron, zinc, and copper level of pregnant women in the study area

5 ml of serum was pipetted into a test tube, and an equal volume of a 10% solution of TCA (tri-chloro-acetic acid) was added. Para film was used to cover the tops of the tubes, which, after vortex-mixing, were left to stand for 10 min, and the tubes were centrifuged. Then the supernatant was removed by Pasteur pipette and placed in another tube from which it was aspirated. Zinc, iron, and copper determinations could be performed on the acidic solution. Stock standards of Cu, Zn, and Fe were prepared by dissolving (15.08 mmol) Cu (NO_3_)_2_, (14.56 mmol) Zn (NO_3_)_2,_ and (0.02 mmolL^−1^) (NH_4_)_2_Fe (SO_4_)_2_·6H_2_O in 1L of distilled water. Then deliver 1 mL of stock standards of Zn, Fe, and Cu into five different sets of working standards containing 50, 100, 200, 300, and 400mmolL^−1^ which were diluted in deionized water for the calibration curve. Finally, the results were analyzed by graphite furnace atomic absorption spectroscopy (GFAAS)^56^. In this study, ferritin serum concentrations in pregnancy were defined as less than ≥ 10 μgL^−1^ and were considered the safe range, and levels less than 10 μgL^−1^ were considered iron deficiency^57^. The serum zinc levels greater or equal to 51 μgdL^−1^ were considered a safe range, while levels less than 51 μgdL^-−1^ were considered as zinc deficiency^58^. Finally, serum copper levels greater or equal to 70 μgdL^−1^ were considered a safe range, while serum levels less than 70 μgdL^−1^ were classified as copper deficiency^59^.

### Data processing and analysis

Data will be entered using EPI-INFO version 7 software and analysis will carry out using SPSS software version 24.0 (IM Corp., Armonk, NY, US). We used percentage, frequency, chi-square test, bi-variate and multivariate analyses to examine the serum metallic level and associated risk factors of pregnant women small-scale farmers. Only elements exhibiting a significant correlation (*p* < 0.25) with each dependent variable in the bi-variate analysis were chosen and adjusted in the multivariate logistic regression analysis. The independent variables were checked for multicollinearity before being included in the final regression model. The variance inflation factor (VIF) was used to assess multicollinearity among independent variables. The strength of the link was measured using an adjusted odds ratio (AOR) with 95% confidence intervals.

### Operational definitions

Iron deficiency: Serum iron concentration deficiency was defined as a serum iron level of less than 10 μgL^−1^ during pregnancy of women’s small-scale farmers^57^.

Zinc deficiency: Serum zinc concentration deficiency was defined as a serum zinc level of less than 51 μgdL^−1^ during pregnancy of women’s small-scale farmers^60,61^.

Copper deficiency: Serum copper concentration deficiency was defined as a serum copper level of less than 70 μgdL^−1^ during pregnancy of women’s small-scale farmers^59^*.*

*Dietary diversity score (DDS)*: The minimum dietary diversity score of pregnant women was classified as low if they consumed less than five food items and higher if they consumed more than or equal to five food items per day^62^.

### Ethical approval

An ethical approval letter was obtained from the Institute of Health Review Board (IRB) of Jimma University on 18/10/2019 (No. IHRPGD/407/2019). The authors confirm that all experiments were performed under relevant guidelines and regulations. Written informed consent was obtained from each study subject.

### Consent to participate

All the participants voluntarily participated.

## Data Availability

The datasets analysed during the current study were available from the corresponding author upon reasonable request.
